# Composition and Functions of the Gut Microbiome in Pediatric Obesity: Relationships with Markers of Insulin Resistance

**DOI:** 10.3390/microorganisms9071490

**Published:** 2021-07-13

**Authors:** Camila E. Orsso, Ye Peng, Edward C. Deehan, Qiming Tan, Catherine J. Field, Karen L. Madsen, Jens Walter, Carla M. Prado, Hein M. Tun, Andrea M. Haqq

**Affiliations:** 1 Human Nutrition Research Unit, Department of Agricultural, Food and Nutritional Science, 4-002 Li Ka Shing Centre for Health Innovation, University of Alberta, Edmonton, AB T6G 2E1, Canada; orsso@ualberta.ca (C.E.O.); carla.prado@ualberta.ca (C.M.P.); 2 HKU-Pasteur Research Pole, School of Public Health, University of Hong Kong, Hong Kong 999077, China; pengye@connect.hku.hk; 3 Department of Medicine, University of Alberta, Edmonton, AB T6G 2C2, Canada; deehan@ualberta.ca (E.C.D.); karen.madsen@ualberta.ca (K.L.M.); 4 Department of Pediatrics, University of Alberta, Edmonton, AB T6G 2R3, Canada; qtan3@ualberta.ca; 5 Department of Agricultural, Food and Nutritional Science, University of Alberta, Edmonton, AB T6G 2E1, Canada; cjfield@ualberta.ca; 6 APC Microbiome Ireland, School of Microbiology, and Department of Medicine, University College Cork—National University of Ireland, T12 YT20 Cork, Ireland; jenswalter@ucc.ie

**Keywords:** gut microbiome, microbiota, shotgun metagenomics, insulin resistance, HOMA-IR, childhood obesity

## Abstract

The gut microbiome is hypothesized to play a crucial role in the development of obesity and insulin resistance (IR); the pathways linking the microbiome to IR in pediatrics have yet to be precisely characterized. We aimed to determine the relationship between the gut microbiome composition and metabolic functions and IR in children with obesity. In a cross-sectional study, fecal samples from children with obesity (10–16 years old) were collected for taxonomical and functional analysis of the fecal microbiome using shotgun metagenomics. The homeostatic model assessment for insulin resistance (HOMA-IR) was determined using fasting glucose and insulin. Associations between HOMA-IR and α-diversity measures as well as metabolic pathways were evaluated using Spearman correlations; relationships between HOMA-IR and β-diversity were assessed by permutational multivariate analysis of variance. Twenty-one children (nine males; median: age = 12.0 years; BMI z-score = 2.9; HOMA-IR = 3.6) completed the study. HOMA-IR was significantly associated with measures of α-diversity but not with β-diversity. Children with higher HOMA-IR exhibited lower overall species richness, Firmicutes species richness, and overall Proteobacteria species Shannon diversity. Furthermore, HOMA-IR was inversely correlated with the abundance of pathways related to the biosynthesis of lipopolysaccharides, amino acids, and short-chain fatty acids, whereas positive correlations between HOMA-IR and the peptidoglycan biosynthesis pathways were observed. In conclusion, insulin resistance was associated with decreased microbial α-diversity measures and abundance of genes related to the metabolic pathways. Our study provides a framework for understanding the microbial alterations in pediatric obesity.

## 1. Introduction

Childhood obesity is commonly associated with an impaired metabolic profile characterized by insulin resistance, dyslipidemia, and low-grade inflammation [1,2,3,4,5]. In fact, insulin resistance is a risk factor for youth-onset type 2 diabetes mellitus (T2DM) and cardiovascular diseases [6]. Several mechanisms have been proposed to explain the pathogenesis of insulin resistance in the pediatric population, including an unfavorable lipid partitioning profile [7], early age of puberty onset [8], a family history of T2DM [9], and less healthy lifestyle choices, including suboptimal dietary intake and low physical activity [10,11]. The homeostasis model assessment (HOMA) is a widely used surrogate measure of insulin resistance that has been validated in children and adolescents against more established techniques, such as the euglycemic clamp and frequently sampled intravenous glucose tolerance test [12,13]. HOMA-IR is calculated using fasting glucose and insulin concentrations as previously described [14]. Although several studies have attempted to define cut-off values for insulin resistance (ranged from 2.5 to 4.0), there is a lack of consensus on the optimal value that should be applied across all pediatric populations [14,15,16,17,18,19].

Increasing evidence points toward the gut microbiome as an important modulator of both obesity and insulin resistance [20,21,22,23]. Animal experiments have established an involvement of gut microbiome in weight gain, adiposity, inflammation, and metabolic diseases through the interaction with dietary components [24,25]. A diet poor in fiber intake in particular, is associated with suboptimal production of microbial-derived short-chain fatty acids (SCFAs), limiting the beneficial secretion of anorexigenic hormones, anti-inflammatory cytokines and mucin in the protective intestinal mucus layer [26,27,28]. Studies in humans have shown marked reductions in gut microbiota diversity in adults with obesity and metabolic abnormalities [29,30]. In some studies, an abundance of specific bacterial species, such as *Prevotella copri* and *Bacteroides vulgatus*, were found to drive the associations between insulin resistance and the biosynthesis of key metabolites (e.g., branched-chain amino acids (BCAA), tryptophan, and lipopolysaccharides (LPS)) implicated in metabolic disease [23,31]. Therefore, shifts in gut microbiota composition during growth and development could alter the symbiotic relationship between gut bacteria and the human host, contributing to the development of obesity-related comorbidities.

While numerous associations between the gut microbiome and insulin resistance in adults have been documented, there remains a lack of understanding of how gut microbiota may be involved in the development of metabolic diseases in the pediatric population [32,33]. Measures of α-diversity have been used to estimate microbial variability in terms of richness and evenness within a sampled community [34,35]. For instance, children with abnormalities of glucose metabolism have been shown to have either lower [36,37], higher [38], or similar microbial α-diversity [39] compared to metabolically healthy children. After following children of normal weight for four years prospectively, a recent study showed that those children who had a low microbial diversity profile and unhealthy diet were more predisposed to obesity and metabolic inflammation than those with a higher microbial diversity [40]. However, it remains unclear whether these microbial imbalances were a causal factor in the development of obesity. Moreover, many studies have profiled the gut microbiota of children and adolescents using 16S rRNA gene amplicon sequencing to compare taxonomic composition across weight status [41,42,43,44], but only a few have explored the associations between markers of insulin resistance and gut microbiome functions [36,37,38,45,46]. None of these studies have used shotgun metagenomics, which is a more robust approach allowing researchers to catalogue all genes present in a sample, identify the taxonomic composition at a species level resolution, and characterize the functional potential of the gut microbiome [47]. Thus, the use of shotgun metagenomics in our study may help to advance the understanding of associations between microbiome composition, functions, and human metabolic diseases. The main objective of the present study was to investigate the relationship of the gut microbiome composition and functions, as determined by shotgun metagenomics, with insulin resistance in children and adolescents with obesity. In addition, we also investigated relationships between the gut microbiome, microbially derived metabolites, clinical characteristics, dietary intake, and physical activity to explore potential mechanisms by which the gut microbiome may influence clinical outcomes.

## 2. Materials and Methods

### 2.1. Study Sample

This study was a cross-sectional study including twenty-one children aged 10 to 16 years with a body mass index (BMI) at or above the 95th percentile for age and sex (equivalent to a BMI z-score of ≥1.64) [48,49]. Exclusion criteria included children with a diagnosis of conditions associated with impaired muscle mass, chronic diseases (e.g., diabetes, chronic liver disease) leading to obesity, acute infections, being pregnant or lactating, taking medications known to influence body composition or antibiotics (two months prior to the study visit), or taking probiotic or dietary fiber supplements for three weeks prior to the study visit. Between August 2018 and March 2020, participants were recruited from the Pediatric Centre for Weight and Health (PCWH) and the surrounding pediatric community in Edmonton (AB, Canada) by either direct contact with the research team or via study ads (i.e., posters, flyers, animated video). A total of 99 children and adolescents were screened for eligibility, but only 21 of those who met the inclusion criteria agreed to provide stool samples. This study was approved by The University of Alberta Health Research Ethics Board (Pro00082135); written parental consent and written child assent were obtained from all participants.

### 2.2. Clinical Assessments

Participants attended two study visits held at least seven days apart (median (interquartile range, IQR) = 11 (9–14) days) at the University of Alberta Human Nutrition Research Unit. Demographic characteristics, medical history information, and self-reported puberty stage were collected at visit 1. Dietary intake and physical activity were assessed at home using a three-day dietary record and accelerometer, respectively. Anthropometry, body composition (by air-displacement plethysmography), and blood pressure were measured at visit 2 (see the Appendix A for additional information).

### 2.3. Blood Sampling and Biochemical Analysis

At visit 2, blood samples were collected after a 12-h overnight fast into silicone-separator gel and EDTA tubes; serum and plasma aliquots were then isolated respectively and stored at −80 °C until further analysis. Serum glucose was measured with an enzymatic hexokinase method (lower limit of detection [LLOD]: 0.2 mmol/L) and C-reactive protein (CRP (inflammatory marker); LLOD: 0.5 mg/L) with an immunoassay method using a clinical chemistry analyzer (Siemens Atellica system). Plasma insulin was also evaluated using an immunoassay method with the Abbott Architect analyzer (LLOD: 7.2 pmol/L). Other inflammatory markers, such as plasma concentrations of interleukin-6 (IL-6; R&D Systems Quantikine, Minneapolis, MN, USA; LLOD: 0.7 pg/mL) and tumor necrosis factor alpha (TNF-α, R&D Systems Quantikine, Minneapolis, MN, USA; LLOD: 6.23 pg/mL) [50], were assessed by ELISA. As a measure of intestinal barrier function, we also evaluated LPS (Abbexa Ltd., Cambridge, UK; LLOD: <0.005 EU/mL), and lipopolysaccharide-binding protein (LBP; USCN Life Science and Technology, Huston, USA; LLOD: 1.21 ng/mL) using ELISA [51]. Glucose and insulin were used to calculate the homeostatic model assessment of IR (HOMA-IR = fasting insulin (mU/mL) × fasting glucose (mmol/L)/22.5) as a marker of glucose metabolism [13,14]. One participant was not able to complete the blood draw at the time of study but had blood work done within 11 days of visit 2 in the same core laboratory for medical purposes; data on glucose and insulin were therefore extracted from the participant’s electronic medical record.

### 2.4. Stool Sampling

Stool samples were collected at home using a collection tube with stabilizer (OMNIgene GUT; OMR-200 kit; DNA Genotek, Ottawa, ON, Canada) [52], according to the manufacturer’s instructions. Participants were advised to collect one stool sample at any time during the week between study visits and store the collection tube at room temperature. Samples were brought to the research unit at visit 2 and transferred to labelled cryovials within 60 days of collection according to manufacturer’s instructions, which were then stored at −80 °C until further analysis. Samples were sent frozen to Microbiome Insights Inc. (Vancouver, BC, Canada) following standardized procedures for the quantification of fecal SCFAs and shotgun metagenomic analyses.

### 2.5. Short Chain Fatty Acid Quantification

Fecal SCFA concentrations were determined using gas chromatography (Thermo Trace 1310, Thermo Fischer Scientific, Waltham, MA, USA) with a flame ionization detector following a protocol adapted from Zhao et al. [53]. Briefly, stool samples were first diluted in MilliQ-grade water and homogenized for 1 min at 4.0 m/s using the FastPrep instrument (MP Biomedicals, Santa Ana, CA, USA). Hydrochloric acid (5M) was then added to a final pH of 2.0. The samples were next centrifuged at 10,000× rpm. Supernatants were then analyzed for SCFAs by gas chromatography. Concentrations of acetate, propionate, butyrate, valerate, isobutyrate, isovalerate were normalized to the amount of input material (SCFA (in mmol)/fecal content (in kg)).

### 2.6. Stool DNA Extraction and Shotgun Metagenomic Sequencing

DNA was extracted from homogenized samples using a commercial extraction kit (Qiagen MagAttract PowerSoil DNA) optimized for the Thermo Scientific KingFisher system, according to manufacturer’s instructions. Sequencing libraries were prepared using the Nextera XT DNA Library Preparation Kit (Illumina, San Diego, CA, USA). Shotgun metagenomic sequencing was performed on a NextSeq 500 System with a paired-end 150-bp protocol in medium-output mode. Metagenomic reads were processed with the Sunbeam pipeline [54] and the quality of reads was evaluated using FastQC v0.11.5 [55]. In brief, adapter sequences were removed with cutadapt v2.6 [56], trimming was conducted using Trimmomatic v0.36 [57], low-complexity reads were identified and discarded by Komplexity v0.3.6 [54], and host-derived sequences were identified, based on the human genome (Genome Reference Consortium Human Reference 37) and removed from downstream analyses. An average of 17.9 M ± 2.5 M reads (mean ± SD) (2.4 G ± 0.4 G bases) remained per sample. The quality-controlled reads were taxonomically classified by MetaPhlAn3 [58]. Functional potential of microbial communities was then evaluated using HUMAnN3 [59] based on the MetaCyc Metabolic Pathway Database [60]. Carbohydrate-Active enZYmes (CAZy) [61] were also analyzed as previously described [62]. α-Diversity (Shannon diversity and species richness) and β-diversity (Bray−Curtis dissimilarity) indices of the taxonomic composition and functional potential were calculated, based on bacterial species and pathway abundance, respectively.

### 2.7. Statistical Analysis

Continuous data (i.e., clinical characteristics, metabolic and inflammatory markers, and SCFAs) are described using median and interquartile range (IQR, 25th–75th percentile) due to the small sample size; categorical data are described using percentage. Differences of continuous variables and categorical variables between sex were evaluated using the Mann Whitney U-test and the Fisher’s exact test, respectively. As several HOMA-IR cut-off points have been proposed for insulin resistance in children and adolescents [15] with no consensus reached for the optimal cut-off point, we categorized participants into three subgroups using data-driven tertiles of HOMA-IR as follows: lowest tertile (HOMA-IR ≤ 2.87), middle tertile (HOMA-IR between 2.87 and 3.94), and highest tertile (HOMA-IR ≥ 3.94). The use of tertiles as previously described [63,64,65], allowed for the variability in microbial composition and functions to be investigated across the entire spectrum of insulin resistance levels. Differences between HOMA-IR tertiles were analyzed using Kruskal−Wallis test, and post hoc comparisons were performed using Dunn’s test with Benjamini−Hochberg correction (continuous variables) or the Fisher’s exact test (categorical variables). The correlations between HOMA-IR (as a continuous variable) and participants’ characteristics were assessed using Spearman correlations (r_s_).

Factors influencing the β-diversity of the microbiome were identified by permutational multivariate analysis of variance (PERMANOVA, using the *vegan* package in R) and principal coordinates analysis (PCoA). Canonical correspondence analysis (CCA) was used to study the relationship between β-diversity and diet, physical activity, inflammation markers, gut barrier markers, and SCFAs. Correlations between HOMA-IR and α-diversity, bacterial species abundance, and metabolic pathway abundance were assessed using Spearman correlations. Partial or semipartial Spearman correlation tests were conducted as sensitivity analysis controlling for age. Dunn’s test with the Benjamini−Hochberg correction was used for comparisons between three groups. Correlations between abundance of selected species and pathways were tested using Spearman correlation test. We performed similar models with CRP as an exploratory outcome in association analyses. Statistical analyses were conducted using R software (v.3.6.1), and the threshold for statistical significance was set at α ≤ 0.05.

## 3. Results

### 3.1. General Characteristics

Twenty-one children (9 males and 12 females; median age = 12.0 (IQR, 10.8–13.3) years) were included in the analysis. Children had a median BMI z-score of 2.9 (IQR, 2.4–3.5) and HOMA-IR of 3.6 (IQR, 2.4–4.4). Most were Caucasian (66.7%), delivered vaginally (66.7%), and exclusively breastfed during infancy (52.4%). While 52.4% of children were at pre−early puberty, 47.6% were at more advanced pubertal stages (i.e., mid−late puberty). Only two children (9.5%) met the recommendations for moderate and vigorous physical activity, and two other children (9.5%) met the recommendations for dietary fiber intake. There were no significant differences between males and females in terms of HOMA-IR, body composition, and inflammatory markers (Table S1).

Correlation coefficients for the relationships between HOMA-IR and clinical characteristics, dietary intake, physical activity, and gut metabolites are reported in Table S2. HOMA-IR was correlated with age (r_s_ = 0.82, *p* < 0.001), sedentary behavior (r_s_ = 0.54, *p* = 0.012), time spent in light physical activity (r _s_ = −0.54, *p* = 0.011), and fat mass index (r_s_ = 0.54, *p* = 0.013). However, HOMA-IR was not significantly correlated with inflammatory markers or SCFAs. Comparisons between HOMA-IR tertiles revealed that children in the highest HOMA-IR tertile were in fact older and at more advanced pubertal stages (i.e., mid−late puberty) than children in the lowest tertile (i.e., pre−early puberty) (Table 1). Although no differences between tertiles were observed for BMI z-score, children in the highest HOMA-IR tertile had greater fat mass and fat-free mass indices. Furthermore, they spent less time in light physical activity and greater time in sedentary activities compared to children in the lowest HOMA-IR tertile. We did not observe any significant intergroup differences in the inflammatory markers or SCFAs.

### 3.2. Fecal Microbiome Composition

#### 3.2.1. Diversity Patterns

**Figure 1:**
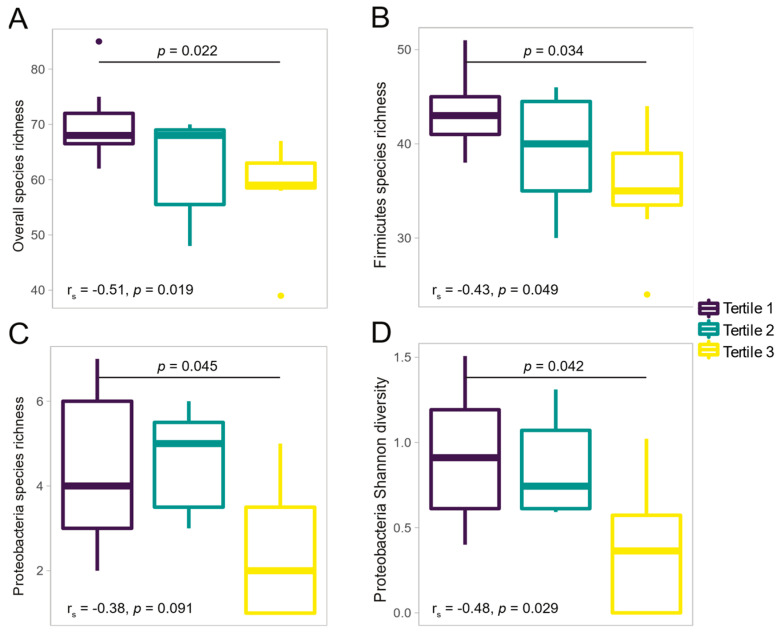
Boxplots of α-diversity indices stratified by homeostatic model assessment for insulin resistance (HOMA-IR) tertiles (N = 21). (**A**) Overall species richness; (**B**) Firmicutes species richness; (**C**) Proteobacteria species richness; and (**D**) Proteobacteria Shannon diversity. Comparisons between HOMA-IR tertiles were performed using the Dunn’s test with Benjamini−Hochberg correction.

We found that HOMA-IR was negatively correlated with overall species richness (r_s_ = −0.51, *p* = 0.019), Firmicutes species richness (r_s_ = −0.43, *p* = 0.049), and Proteobacteria Shannon diversity (r_s_ = −0.48, *p* = 0.029). Only the relationship between HOMA-IR and Shannon diversity of Proteobacteria species (r_s_ = −0.59, *p* = 0.006) remained significant when controlling for age (Table S3). Comparisons between subgroups revealed that children in the highest HOMA-IR tertile had lower species richness than those in the first tertile (*p* = 0.022) (Figure 1). Differences in the number of observed species were identified particularly for the Firmicutes (*p* = 0.034) and Proteobacteria (*p* = 0.045) phyla. Furthermore, children in the highest HOMA-IR tertile also exhibited the lowest Shannon diversity for the Proteobacteria phylum (*p* = 0.042). In contrast to the α-diversity findings, HOMA-IR was not associated with measures of β-diversity at the species level using PERMANOVA.

In the exploratory analysis, species richness correlated with age (r_s_ = −0.44, *p* = 0.046), sedentary time (r_s_ = −0.63, *p* = 0.002), and time spent in light (r_s_ = 0.54, *p* = 0.013) and moderate-to-vigorous (r_s_ = 0.47, *p* = 0.030) physical activity; however, no associations with body composition and inflammatory markers were observed. We found that the overall Shannon diversity was negatively correlated with CRP independent of age (r_s_ = −0.46, *p* = 0.046), but this inflammatory marker did not correlate with measures of β-diversity. Furthermore, positive associations were identified between β-diversity and SCFAs (particularly propionate and acetate [R^2^ = 0.19, *p* = 0.001]), adjusted fiber intake (R^2^ = 0.13, *p* = 0.030), BMI z-score (R^2^ = 0.11, *p* = 0.041) as well as reports of jaundice at birth (R^2^ = 0.19, *p* = 0.013). Using CCA, we found that these associations could be explained by variability in the diversity of several species; the top five species (with the lowest *p*-values) were *Prevotella. copri, Paraprevotella xylaniphila, Lachnospira pectinoschiza, Paraprevotella clara,* and *Faecalibacterium prausnitzii* (Table S4).

#### 3.2.2. Differentially Abundant Species

The composition of the gut bacterial community in the total study cohort was dominated by the phyla Bacteroidetes (median abundance = 69.5%), Firmicutes (23.3%), Actinobacteria (0.6%), and Proteobacteria (0.3%). Nevertheless, there were no significant differences in the median abundance of these phyla across HOMA-IR tertiles (Figure S1A). The five most abundant bacterial species (based on median values) in the total study cohort were *B. vulgatus* (6.7%), *F. prausnitzii* (6.4%), *Bacteroides uniformis* (3.8%), *Alistipes putredinis* (3.3%), and *Bacteroides caccae* (1.2%); however, these species did not differ in median abundance between HOMA-IR tertiles (Figure S1B).

The abundance of the five species was negatively correlated with HOMA-IR, including two bacterial species related to butyrate production (*Oscillibacter* sp. *CAG 241* (r_s_ = −0.62, *p* = 0.002), *Agathobaculum butyriciproducens* (r_s_ = −0.46, *p* = 0.037)) and three Gram-negative species (*Haemophilus parainfluenzae* (r_s_ = −0.56, *p* = 0.008), *Veillonella parvula* (r_s_ = −0.53, *p* = 0.013), and *Dialister invisus* (r_s_ = −0.50, *p* = 0.020)). After adjusting for the effects of age, only the correlations between HOMA-IR and *Oscillibacter* sp. *CAG 241* (r_s_ = −0.59, *p* = 0.006) as well as *H. parainfluenzae* (r_s_ = −0.45, *p* = 0.049) remained significant. Children in the highest HOMA-IR tertile had lower abundance of *Oscillibacter* sp. *CAG 241* (*p* = 0.004), *V. parvula* (*p* = 0.032), and *D. invisus* (*p* = 0.021). Furthermore, the Firmicutes to Bacteroidetes (F/B) ratio and *Prevotella* to *Bacteroides* (P/B) ratio were not associated with HOMA-IR (Table S2). In exploratory analysis, abundance of eleven species correlated negatively with CRP levels (Figure S2); however, positive correlations of CRP to bacterial species related to propionate (*Bacteroids eggerthii*; r_s_ = 0.49, *p* = 0.030) and butyrate production (*Anaerotruncus* sp. CAG 528; r_s_ = 0.46, *p* = 0.042) were identified.

### 3.3. Fecal Microbiome Functions

#### 3.3.1. Diversity Patterns

**Figure 2:**
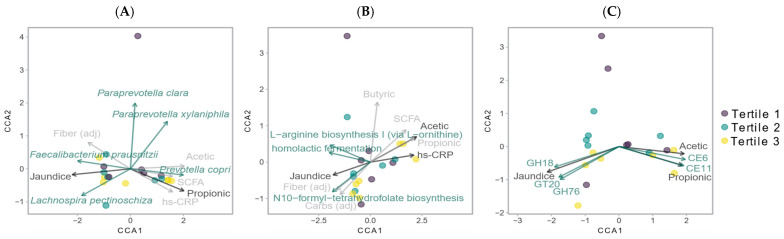
Canonical correspondence analysis showing drivers of β-diversity of (**A**) microbial composition, (**B**) MetaCyc pathways and (**C**) CAZymes.

We found that HOMA-IR was not associated with α-diversity and β-diversity of the MetaCyc pathways, while CRP was negatively correlated with the Shannon diversity of these pathways (r_s_ = −0.63, *p* = 0.004, semipartial correlation adjusting for age). In the exploratory analysis, we observed that β-diversity of the MetaCyc pathways was associated with obesity indices (BMI z-score (R^2^ = 0.26, *p* = 0.004), and fat mass index (R^2^ = 0.25, *p* = 0.003)), total SCFAs (R^2^ = 0.24, *p* = 0.006), acetate (R^2^ = 0.36, *p* = 0.001), propionate (R^2^ = 0.32, *p* = 0.001), and CRP (R^2^ = 0.32, *p* = 0.006). Canonical correspondence analysis showed that approximately 52% of the identified pathways were significant drivers of β-diversity, including those ones related to the metabolism of BCAA (r_s_ range: 0.49–0.96, all *p* ≤ 0.011) (Figure 2B).

#### 3.3.2. Differentially Abundant Pathways

**Figure 3:**
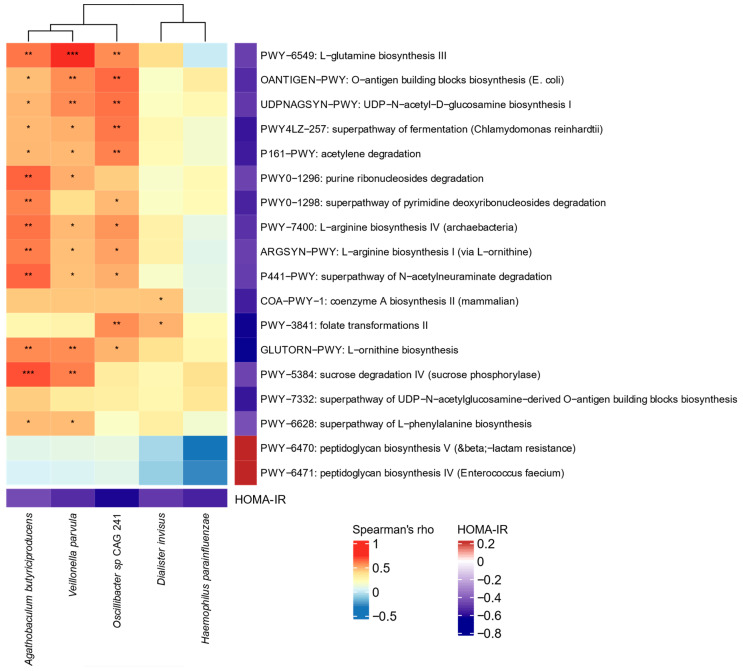
Heat map showing correlations of gut microbiome composition and functions with the homeostatic model assessment for insulin resistance (HOMA-IR) in 21 children with obesity (unadjusted analysis).

We identified 18 pathways related to either metabolic or biological functions of the gut microbiome to be significantly associated with HOMA-IR (Figure 3 and Figure S3). Of these, HOMA-IR was negatively correlated with pathways linked to arginine, glutamine, and phenylalanine biosynthesis. Although there were no significant associations between HOMA-IR and circulating LPS and LBP (Table S2), negative correlations were observed between HOMA-IR and pathways for LPS. Higher HOMA-IR also correlated with a lower abundance of pathways related to folate biosynthesis, pyruvate, and coenzyme A as well as SCFAs production.

Conversely, HOMA-IR was positively correlated with pathways related to bacteria cell wall biosynthesis, particularly of peptidoglycans. Most of these correlations remained significant after including age as a covariate (Table S3). Furthermore, we observed that eleven out of the eighteen pathways were reduced in children in the highest HOMA-IR tertile; however, children in the third tertile had the greatest abundance of a pathway related to bacteria cell wall synthesis. Because there is evidence supporting the role of BCAA in the development of insulin resistance [31,66], and we detected an association between BCAA-related pathways and β-diversity, we also examined whether BCAA pathways differed between HOMA-IR tertiles; however, no differences were found. In the exploratory analysis, an abundance of 89 MetaCyc pathways were correlated with CRP (Figure S2).

### 3.4. Carbohydrate-Active Enzyme (CAZyme) Analysis

CAZyme β-diversity was not shown to be associated with HOMA-IR. In contrast, significant associations between CAZymes β-diversity and lipopolysaccharide-binding protein (LBP) (R^2^ = 0.14, *p* = 0.043), SCFAs (especially acetate (R^2^ = 0.13, *p* = 0.044), and propionate (R^2^ = 0.13, *p* = 0.047)) were observed, although neither of these associations remained significant after *p* value correction. Canonical correspondence analysis showed that associations between β-diversity and SCFAs were driven by enzymes from the carbohydrate esterase families 6 and 11; in contrast, associations between β-diversity and jaundice at birth may be explained by the glycoside hydrolase families 18 and 76 as well as the glycosyltransferase family 20 (Figure 2C). In addition, the total abundance of CAZyme families was not different between HOMA-IR tertiles (Figure S4).

## 4. Discussion

This study in children with obesity shows that the β-diversity of the gut microbiome was not associated with HOMA-IR, consistent with previous studies in adolescents and adults [45,67]. The overall microbial α-diversity (using the Shannon diversity) has been reported to be negatively correlated with HOMA-IR [36] or type 1 diabetes mellitus status in children [37]. We identified that species richness, but not overall α-diversity, had a negative association with HOMA-IR. Of note, we report for the first time that children with higher HOMA-IR levels had lower species richness and Proteobacteria species Shannon diversity, independent of age. Particularly, the abundance of *H. parainfluenzae* was negatively correlated with HOMA-IR in our study, which corroborates the findings of Del Chierico et al. in youths with obesity, aged 9 to 18 years [68].

Previous research showed a detrimental role of bacterial LPS translocation (termed endotoxemia) on insulin sensitivity by activating Toll-like receptors 4 (TLR4) and triggering the secretion of proinflammatory cytokines [36,69,70,71]. Our study demonstrated negative correlations between HOMA-IR and the abundance of genes associated with LPS biosynthesis pathways. Specifically, we showed the downregulation of the UDP-N-acetyl-d-glucosamine pathway in children with high HOMA-IR (Figure 3), which is involved in the synthesis of the lipid A structure of LPS that binds to TLR4 [72]. We therefore speculate that there may be other altered functional attributes of the gut microbiome likely initiating inflammatory responses in young individuals.

In our study, positive correlations were found for HOMA-IR with two pathways related to the biosynthesis of peptidoglycans. This is relevant in light of emerging evidence suggesting that these bacteria cell-wall components, when sensed by multiple pattern-recognition receptors, have also been shown to contribute to insulin resistance through modulating the immune response, increasing the translocation of bacterial components, and promoting vascular and adipose tissue inflammation [70,73,74,75,76,77,78]. However, future studies are required to better elucidate mechanisms regulating the interactions between peptidoglycans metabolites and changes in metabolism during childhood.

Another functional attribute of the gut microbiome related to insulin resistance is amino acid metabolism. Prior studies have revealed beneficial immunomodulatory effects of some amino acids; for example, glutamine and arginine can lower inflammation and are precursors for the adequate functioning of neutrophils, macrophages, and enterocytes [79]. Remarkably, acute treatment with *l*-glutamine enhanced insulin-response and increased glucagon-like peptide-1 (GLP-1) in adults with well-controlled T2DM [80]. Thus, children with higher HOMA-IR in our study might not have benefited from bacterial production of amino acids as they presented with lower abundance of genes linked to amino acid biosynthesis. Despite these beneficial roles, metabolomics studies have consistently reported strong associations between circulating BCCA levels and insulin resistance in diverse populations [31]. Our study did not find such associations, but the abundance of BCAA pathways were positive drivers of β-diversity of the MetaCyc pathways, which was associated with obesity indices and inflammation.

The inflammatory processes have been shown to be altered in children with obesity and associated with the development of insulin resistance [5,81]. In our exploratory analyses to examine the relationships between the gut microbiome and inflammation, we found that taxonomic α-diversity (overall Shannon diversity) reduced with increases in CRP in children with obesity. This observation aligns with previous studies in the adult population, as reported in a systematic review and confirmed in a large scale study published recently [82,83]. Negative associations were also found between CRP and abundance of several SCFAs producers, suggesting the beneficial immune response to these bacterial metabolites. In contrast, we showed that abundance of *B. eggerthii* (which has been shown to form propionate in the presence of vitamin B_12_ [84]) was positively correlated with CRP levels. Although we did not evaluate SFCAs from plasma samples, one study showed significant positive associations between circulating SCFAs and adiposity measurements in adolescents [85]. Coupled with these findings, it was demonstrated that the microbiota from adolescents with obesity had a greater ability to ferment carbohydrates than those of normal weight. However, our study showed a similar capacity of the gut microbiota with regard to carbohydrate metabolism in children with and without insulin resistance as the diversity and abundance of CAZymes did not differ across HOMA-IR tertiles. These findings might be explained by the lack of a relationship between Bacteroidetes and insulin resistance, as this phylum encodes more diverse CAZymes than other phyla of gut microbiota [86].

In this study, we used shotgun metagenomic analysis, and thus were able to profile and compare taxonomic compositions at a species-level resolution, as well as profile functional potentials of the microbiome more accurately than estimates from 16S rRNA gene amplicon sequences [47]. We also evaluated a series of factors previously shown to be determinants of metabolic health using reference techniques, such as body composition by air-displacement plethysmography, physical activity using accelerometry, and dietary intake by three-day food records. However, our sample size of 21 individuals limited the ability to adjust the analysis for multiple covariates simultaneously and investigate which of the identified pathways contributed to insulin resistance to the greatest extent. Considering the COVID-19 pandemic, a sufficient number of Canadian children of both sexes were still recruited in a timely manner before research activities were put on hold. Challenges in recruiting children with obesity, specifically those with metabolic abnormalities, are well-documented in several pediatric studies [87,88]. Although we did not enroll children without obesity as controls (i.e., case-control design), our results provide evidence of distinct taxonomic and functional profiles of the gut microbiome across HOMA-IR tertiles even in the presence of obesity. Another limitation of our study is that only a small subset of metabolites was explored. Fecal metabolome, as well as blood metabolome should be evaluated to understand to what extent the microbiota-derived metabolites may affect host physiology. Along the same lines, future studies should consider measuring both plasma and fecal SCFAs, as previous clinical studies have shown that plasma but not fecal SCFAs were associated with markers of insulin sensitivity and the degree of SCFA absorption to circulation is not the same across individuals [89,90].

## 5. Conclusions

**Figure 4:**
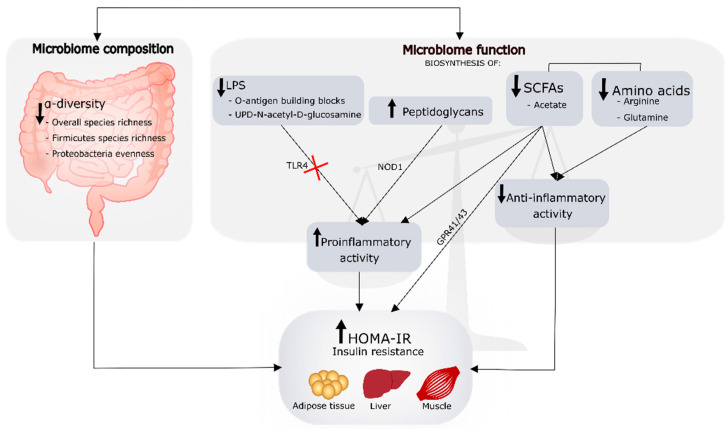
Graphical summary of the altered microbiome composition and functions associated with insulin resistance, determined by the homeostatic model assessment for insulin resistance (HOMA-IR) in the cohort study. Previous evidence has suggested a role of lipopolysaccharides (LPS) on the development of metabolic diseases. Here we found that other functional pathways might be contributing to a greater extent to insulin resistance through imbalances between proinflammatory and anti-inflammatory activities. These pathways are related to the biosynthesis of peptidoglycans, short-chain fatty acids (SCFAs), and amino acids. Potential mechanisms explaining these associations are briefly described in the figure. Abbreviations: TLR4, toll-like receptor 4; NOD1, nucleotide-binding oligomerization domain 1; GPR43, G-protein coupled receptors 43.

Children with obesity and higher HOMA-IR levels, reflecting insulin resistance, exhibited lower α-diversity metrics for Proteobacteria species as well as a lower abundance of bacteria related to butyrate production and Gram-negative bacteria. Notably, our study is one of the few that have examined the functional potentials of gut bacteria communities in children with obesity (Figure 4). We further reported that higher HOMA-IR levels were also associated with a lower abundance of amino acids and SCFAs biosynthesis pathways, possibly limiting the beneficial contribution of these metabolites to insulin sensitivity. Moreover, positive associations between HOMA-IR and the pathways related to peptidoglycan biosynthesis are in line with previous mechanistic studies suggesting a role of these cell-wall components to the development of insulin resistance. While findings should be interpreted with caution due to the limited sample size, our study provides a framework for future investigations on: (i) the mechanisms regulating the interactions between peptidoglycans metabolites and changes in metabolism; and (ii) the taxonomic drives of functional imbalances associated with glucose metabolism abnormalities in children with obesity (using both metagenomics and metabolomics). Furthermore, prospective cohort studies evaluating children at high risk for T2DM during pubertal growth are extremely valuable in depicting factors that determine the progression of insulin resistance to T2DM. Collectively, these studies may inform interventions targeting both bacterial communities and their functional attributes, with the potential to improve the efficacy of pediatric obesity and metabolic disease treatment.

## Figures and Tables

**Table 1:** Participants’ characteristics stratified by data-driven tertiles of the homeostatic model assessment for insulin resistance (HOMA-IR) (N = 21).

	HOMA-IR			*p*-Value *
	Lowest Tertile (n = 7)	Middle Tertile (n = 7)	Highest Tertile (n = 7)	
Age (years)	10.7 (10.2–10.9)	12.1 (11.7–13.0) ^a^	13.4 (13.2–15.6) ^b^	0.001
Sex (% male)	4 (57.1)	3 (42.9)	2 (28.6)	0.854
**Sexual maturation**				
Pre−early puberty (%)	6 (85.5)	4 (57.1)	1 (14.3)	0.040
**Race/ethnicity**				
White (%)	3 (42.9)	7 (100)	4 (57.1)	0.096
Born preterm (%)	1 (14.3)	0 (0)	2 (28.6)	0.742
Vaginal birth (%)	6 (85.7)	3 (42.9)	5 (71.4)	0.381
Exclusively breast fed (%)	4 (57.1)	4 (57.1)	3 (42.9)	1.000
**Dietary intake**				
TEI (kcal/day)	2000 (1790–2010)	2000 (1700–2380)	1660 (1020–2020)	0.310
CHO (g/1000 kcal)	113.6 (108.6–119.8)	132.4 (127.5–136.2) ^a^	124.3 (99.0–133.6)	0.074
Fat (g/1000 kcal)	40.7 (39.5–43.6)	37.0 (35.6–38.7)	35.9 (32.9–42.1)	0.106
Protein (g/1000 kcal)	41.6 (41.3–44.7)	41.5 (36.0–44.2)	45.7 (44.5–52.7)	0.134
Fiber (g/1000 kcal)	8.9 (8.0–9.3)	11.5 (9.9–11.9)	8.3 (7.6–9.8)	0.103
Total fiber intake (g/day)	15.3 (14.3–17.6)	20.6 (18.7–28.5) ^c^	10.2 (7.8–20.5)	0.056
High fiber (%)	1 (14.3)	1 (14.3)	0 (0)	1.000
**Physical activity**				
Sedentary time (min)	488 (470–532)	640 (581–694) ^a^	698 (617–713) ^b^	0.016
Light PA (min)	191 (167–245) ^a,b^	116 (101–143)	112 (100–149)	0.022
MVPA (min)	48 (40–63)	26 (19–39)	34 (27–44)	0.133
Low MVPA (%)	5 (71.4)	7 (100)	7 (100)	0.300
**Anthropometrics and body composition**				
Body weight (kg)	56.1 (53.6–70.9)	78.8 (59.2–90.6)	101.6 (79.1–118.0) ^b,c^	0.020
BMI z-scores	2.5 (2.0–3.4)	2.9 (2.5–3.1)	3.3 (2.8–4.0)	0.382
%BF, males/females (%)	40.9 (39.4–42.9)/38.9 (33.4–39.9)	42.5 (36.4–48.6)/42.4 (39.5–45.0)	39.5 (37.0–41.9)/50.3 (45.3–53.5)	0.146
FMI, males/females (kg/m^2^)	12.4 (10.4–13.7)/9.3 (7.6–10.0)	11.7 (10.4–17.9)/12.8 (11.3–13.9)	12.4 (11.5–13.3)/18.7 (14.5–25.3) ^b^	0.059
FFMI, males/females (kg/m^2^)	16.4 (15.5–17.3)/15.4 (15.0–15.4)	20.0 (17.9–20.4)/16.5 (15.8–17.7)	18.9 (18.4–19.5)/19.8 (18.5–21.9) ^b^	0.042
**Metabolic parameters**				
Glucose (mg/dL)	86.4 (82.8–90.0)	84.6 (81.9–87.3)	90.0 (90.0–91.80) ^c^	0.035
Insulin (pmol/L)	67.4 (54.9–76.1)	113.2 (109.4–119.5) ^a^	177.1 (155.6–210.1) ^b,c^	>0.001
HOMA-IR	2.20 (1.61–2.32)	3.55 (3.18–3.68) ^a^	5.67 (4.94–6.92) ^b,c^	>0.001
CRP (mg/L)	2.0 (1.4–4.4)	2.2 (0.9–4.3)	9.7 (1.1–14.6)	0.549
IL-6 (pg/mL)	24.0 (6.6–44.1)	13.8 (6.9–51.2)	9.6 (6.0–12.0)	0.748
TNF-α (pg/mL)	27.5 (5.2–71.5)	7.35 (6.8–15.0)	32.10 (5.8–39.2)	0.795
LBP (ug/mL)	15.1 (12.8–39.5)	33.6 (15.9–46.6)	27.4 (22.3–33.1)	0.714
LPS (EU/mL)	0.66 (0.44–0.77)	0.38 (0.33–0.49)	0.48 (0.39–0.93)	0.243
**SCFAs ^†^**				
Acetate (mmol/kg)	35.8 (34.4–38.7)	25.6 (22.7–33.9)	33.9 (24.5–50.9)	0.120
Propionate (mmol/kg)	7.9 (4.8–8.1)	2.8 (0.4–7.1)	7.6 (5.2–12.5)	0.227
Butyrate (mmol/kg)	12.8 (8.5–18.5)	9.4 (7.4–10.2)	9.3 (6.1–13.9)	0.483
Valerate (mmol/kg)	1.1 (0.9–2.0)	0.9 (0.3–1.0)	1.2 (0.9–1.6)	0.246
Isobutyrate (mmol/kg)	2.9 (1.4–3.8)	0.0 (0.0–1.6)	1.0 (0.2–2.1)	0.198
Isovalerate (mmol/kg)	1.9 (1.4–2.6)	0.7 (0.5–1.2)	1.2 (1.0–2.2)	0.198
Total SCFAs (mmol/kg)	55.8 (51.0–80.8)	41.0 (33.4–57.4)	55.8 (41.7–77.7)	0.205

Data is presented using median and interquartile range (25th percentile–75th percentile), except for categoric data shown as count (%). Abbreviations: %BF, percent body fat; CHO, carbohydrate; FFMI, fat-free mass index; FMI, fat mass index; HOMA-IR, homeostatic model assessment of insulin resistance; CRP, C-reactive protein; IL-6, interleukin-6; IQR, interquartile range; LBP, lipopolysaccharide binding protein; LPS, lipopolysaccharides; MVPA, moderate-to-vigorous physical activity; PA, physical activity; SCFAs, short-chain fatty acids; TEI, total energy intake; TNF-α, tumor necrosis factor alpha. ^a^ Significant difference between the lowest and middle HOMA-IR tertiles (*p* < 0.05). ^b^ Significant difference between the lowest and the highest HOMA-IR tertiles (*p* < 0.05). ^c^ Significant difference between the middle and highest HOMA-IR tertiles (*p* < 0.05). * *p*-values for comparisons between HOMA-IR tertiles. Continuous variables were presented as median (interquartile range (IQR)) and were compared using Kruskal−Wallis test, and post hoc comparisons were performed using Dunn’s test with Benjamini−Hochberg correction. Categorical variables were shown as count (%) and were compared using the Fisher’s exact test. ^†^ Short-chain fatty acids (SCFAs) were normalized to the amount of input material (SCFA (in mmol)/fecal content (in kg)).

## Data Availability

All data generated and analyzed during this study are included in this article and its supplementary information files.

## Supplementary Materials

The following are available online at https://www.mdpi.com/article/10.3390/microorganisms9071490/s1, Supplementary methods; Table S1: Participants’ characteristics stratified by sex, Table S2: Correlations between the HOMA-IR and clinical characteristics, dietary intake, physical activity, and gut metabolites, Table S3: Partial correlations adjusted for age between HOMA-IR and α-diversity, species, and pathways, Table S4: Significant contributors of bacterial species β-diversity detected by canonical correspondence analysis, Figure S1: Relative abundance of phyla and species across HOMA-IR tertiles, Figure S2: Heat maps showing correlations of gut microbiota composition and function with CRP, Figure S3: Heat map showing correlations of gut microbiota composition and function with the homeostatic model assessment for insulin resistance, Figure S4: Abundance of CAZymes in children with obesity stratified by data-driven HOMA-IR tertiles.

## Appendix A

### Appendix A.1. Clinical Assessments

#### Appendix A.1.1. Demographics and Clinical Variables

We collected data on age, sex, and race/ethnicity. We classified race/ethnicity into three categories: white, indigenous, and others (e.g., Latino, black, Arabic). Parents completed a medical history questionnaire, reporting child’s gestational age, birth weight at delivery, birth mode, jaundice at birth, and feeding practices during the first year of life. Preterm birth was defined as gestational age less than 37 weeks. Size for gestational age was calculated in PediTools using the 2013 Fenton growth charts [91]. Exclusive breastfeeding was defined as infant receiving only human breast milk for ≥ 6 months since birth [92].

Children completed a self-assessment puberty questionnaire containing descriptions and drawings of genitals, breasts, and pubic hair development stages [93]. Participants were classified into two pubertal groups: pre−early (Tanner stages 1 and 2) and mid−late (Tanner stages 3 to 5) [94].

#### Appendix A.1.2. Anthropometric and Body Composition Assessment

Anthropometric and body composition assessment were conducted by the same trained researcher during study visit 2. Children were asked to abstain from high intensity physical activity for 24 h and water consumption for 4 h prior to the visit. All measurements were taken with children wearing minimal clothing (i.e., a tight-fitting bathing suit) and barefooted, after they had emptied their bladders.

One measurement of body weight was measured to the nearest 0.1 kg using a calibrated scale coupled to the air-displacement plethysmography (ADP, Bod Pod 1SB-060M, Life Measurement Instruments, Concord, CA, USA) equipment. Height was measured twice to the nearest 0.1 cm using a wall-mounted stadiometer. Body mass index z-score was computed using the WHO Anthroplus software (v.1.0.4, Geneva, Switzerland).

Body composition was estimated using the ADP equipment according to the manufacturer’s instructions. Thoracic gas volume was predicted using a standard predictive equation based on age, sex, and height [95]. From body volume measures and density calculations, percent body fat (% BF) was estimated using the Lohman equation and fat-free mass (FFM) was calculated by subtracting fat mass (FM) from total body weight [96]. Fat mass and FFM (in kg) were divided by squared height (in meters) to calculate the FM index (FMI) and FFM index (FFMI), respectively.

#### Appendix A.1.3. Dietary and Physical Activity Assessment

Children assisted by their parents were asked to complete a 3-day dietary record over two weekdays and one weekend day, after receiving instructions on how to record dietary intake and measure food portions. To ensure completeness, a member of the research staff reviewed the dietary records with the children and their parents upon return (visit 2). Average daily macronutrient intake (protein, carbohydrate, and fat) and total energy intake were determined using Food Processor SQL (v. 11.0.124, ESHA Research), with the Canadian Nutrition File database as the main source for obtaining food nutrient content. The United States Department of Agriculture Nutrient database or manufacturer’s food labels were also used when food nutrient content was not available. The average daily intake of macronutrients per 1000 kcal was used to calculate nutrient density [97]. The adequate intake of total fiber was defined using dietary reference intakes specific for age and sex (males aged 9–13 years = 31 g/day; males aged 14–18 years = 38 gm/day; female aged 9–18 years = 26 g/day; Institute of Medicine, 2002) [98].

Physical activity was measured using accelerometry (4MB GT3X, Actigraph, Pensacola, FL, USA), with epoch length set at 5 s. Children were instructed to wear the device on their right hip attached to a belt over seven consecutive days during all waking hours (except while bathing, showering, or swimming). Data was downloaded and screened for compliance using the ActiLife6 software (v.6.13.4; ActiGraph, LLC, Pensacola, FL, USA); those participants with a minimum of 10 h wear time on at least three days were retained for analysis. The Evenson cut-points were selected to categorized accelerometry data into three intensity levels: sedentary behavior, light intensity, and moderate plus vigorous physical activity (MVPA) [99]. Time spent in each category was reported in minutes and as proportion of total wear time. Children spending less than 60 min in MVPA per day were considered as not meeting the physical activity recommendations [100].
