# Supplementary material for: Composition and Functions of the Gut Microbiome in Pediatric Obesity: Relationships with Markers of Insulin Resistance
Source: Microorganisms. 2021 Jul 13;9(7):1490. doi: 10.3390/microorganisms9071490 (PMC8304481; doi:10.3390/microorganisms9071490)
Supplement: Supplementary file 1 [file microorganisms-09-01490-s001.zip › microorganisms-1292601-supplementary.pdf]

Supplementary Material

Composition and functions of the gut microbiome in pediatric obesity: relationships with markers of insulin resistance

Camila E. Orsso [1,†], Ye Peng [2,†], Edward C. Deehan [3], Qiming Tan [4], Catherine J. Field [5], Karen L. Madsen [3], Jens Walter [6], Carla M. Prado [1], Hein M. Tun [2,*], Andrea M. Haqq[4,5*]

[1] Human Nutrition Research Unit, Department of Agricultural, Food and Nutritional Science, University of Alberta, 4-002 Li Ka Shing Centre for Health Innovation, Edmonton, AB, T6G 2E1, Canada; C.E.O., orsso@ualberta.ca; C.M.P., carla.prado@ualberta.ca

[2] HKU-Pasteur Research Pole, School of Public Health, University of Hong Kong, Hong Kong 999077, China; Y.P., pengye@connect.hku.hk; H.M.T., heinmtun@hku.hk

[3] Department of Medicine, University of Alberta, Edmonton, AB, T6G 2C2, Canada; E.C.D., deehan@ualberta.ca; K.J.M., karen.madsen@ualberta.ca

[4] Department of Pediatrics, University of Alberta, Edmonton, AB T6G 2R3, Canada; Q.T., qtan3@ualberta.ca; A.M.H., haqq@ualberta.ca

[5] Department of Agricultural, Food and Nutritional Science, University of Alberta, Edmonton, AB T6G 2E1, Canada; C.J.F., cjfield@ualberta.ca

[6] APC Microbiome Ireland, School of Microbiology, and Department of Medicine, University College Cork – National University of Ireland, Cork, T12 K8AF, Ireland; J.W., jenswalter@ucc.ie

* Correspondence: heinmtun@hku.hk; haqq@ualberta.ca

Supplementary Material

1 Supplementary Tables

Table S1. Participants' characteristics stratified by sex (N = 21).

	Males (n = 9)	Females (n = 12)	p-value*
Age (years)	11.3 (10.2-12.0)	12.9 (11.4-13.9)	0.126
White (%)	5 (55.6)	9 (75.0)	0.550
Pre-early puberty (%)	5 (55.6)	6 (50.0)	1.000
Born preterm (%)	1 (11.1)	2 (16.7)	1.000
Vaginal birth (%)	6 (66.7)	8 (66.7)	1.000
Breast fed (%)	4 (44.4)	7 (58.3)	1.000
**Dietary intake**			
TEI (kcal/day)	1932.9 (1778.1-2006.5)	1853 (1635.4-2165.1)	0.972
CHO (g/1,000 kcal)	123.4 (104-130.9)	126.6 (113.5-133.1)	0.917
Fat (g/1,000 kcal)	37.3 (35.9-40.7)	39.8 (36.2-42.7)	0.754
Protein (g/1,000 kcal)	39.8 (36.2-42.7)	42.3 (40.9-46.2)	0.508
Fiber (g/1,000 kcal)	8.9 (7.8-9.6)	9.7 (8.2-11.6)	0.602
Fiber – total intake (g)	17.6 (15.3-18.1)	17.1 (12.9-24.5)	0.972
High fiber (%)	0 (0)	2 (16.7)	0.486
**Physical activity**			
Sedentary time (min)	537.2 (488.1-628.3)	634.7 (542.3-698.7)	0.345
Light PA (min)	162.7 (122.5-200)	122.7 (105.9-174.9)	0.355
MVPA (min)	48.2 (40.1-49.1)	30.1 (19.5-35.6)	**0.018**
Low MVPA (%)	8 (88.9)	10 (91.7)	1.000
**Anthropometrics and body composition**			
Body weight (kg)	78.3 (68.4-82.2)	74.8 (58-102.4)	0.862
BMI z-scores	3.3 (2.8-3.5)	2.7 (2.2-3.1)	0.310
%BF (%)	41.6 (36.9-44.3)	43.3 (40.1-48)	0.522
FMI (kg/m^2)	11.7 (10.6-14.2)	13.1 (10.3-16.1)	0.508
FFMI (kg/m^2)	17.8 (16.2-20)	16.5 (15.4-19.8)	0.602
**Metabolic parameters**			
Glucose (mg/dL)	86.4 (81-91.8)	88.2 (86-90)	0.775
Insulin (pmol/L)	111.8 (81.3-122.9)	123.3 (93.2-185.1)	0.277
HOMA-IR	3.1 (2.4-3.8)	3.6 (2.9-5.9)	0.345
hs-CRP (mg/L)	2.8 (0.7-5.9)	2.2 (1.4-7.9)	0.698
IL-6 (pg/mL)	9.3 (7-13.9)	12 (5.7-49.6)	0.711
TNF-α (pg/mL)	18.1 (14-39.9)	7.4 (1-41.1)	0.469
LBP (ug/mL)	32.3 (15.6-46.1)	23.2 (13.1-37)	0.482
LPS (EU/mL)	0.4 (0.4-0.7)	0.5 (0.4-0.8)	0.967
**SCFAs†**			
Acetic (mmol/kg)	34 (23.1-39.5)	34.2 (25.4-37.8)	0.862

Supplementary Material

	Males (n = 9)	Females (n = 12)	p-value*
Propionic (mmol/kg)	7.9 (7.2-10.6)	4 (1-8.8)	0.434
Isobutyric (mmol/kg)	0.6 (0-2.6)	1.1 (0-3.1)	0.856
Butyric (mmol/kg)	9.4 (7.8-12.8)	9.4 (6.4-15.3)	0.917
Isovaleric (mmol/kg)	1.2 (0.6-2.3)	1.4 (0.9-2)	0.602
Valeric (mmol/kg)	0.9 (0.6-1.5)	1.1 (0.7-1.4)	0.775
Total SCFAs (mmol/kg)	55.8 (44.8-61)	52.3 (38.2-69.2)	1.000

Continuous data was presented as median (interquartile range (IQR)); categorical variables were shown as count (%).
Abbreviations: %BF, percent body fat; CHO, carbohydrate; FFMI, fat-free mass index; FMI, fat mass index; HOMA-IR, homeostatic model assessment of insulin resistance; hs-CRP, high sensitivity c-reactive protein; IL-6, interleukin-6; IQR, interquartile range; LBP, lipopolysaccharide binding protein; LPS, lipopolysaccharides; MVPA, moderate-to-vigorous physical activity; PA, physical activity; SCFAs, short-chain fatty acids; TEI, total energy intake; TNF-α, tumor necrosis factor alpha.
* P-values for comparisons between sex groups. Continuous variables were compared using Mann-Whitney U-test. Categorical variables were compared using the Fisher's exact test.
† Short-chain fatty acids (SCFAs) were normalized to the amount of input material (SCFA [in mmol] / fecal content [in kg]).

Supplementary Material

Table S2. Spearman's correlation coefficient (rs) for correlations between the homeostatic model assessment of insulin resistance (HOMA-IR) and clinical characteristics, dietary intake, physical activity, and gut metabolites (N=21).

	**HOMA-IR** r_s	**p-value**
Age (years)	**0.82**	**>0.001**
**Dietary intake**		
TEI (kcal/day)	-0.29	0.196
CHO (g/1,000 kcal)	0.16	0.487
Fat (g/1,000 kcal)	-0.41	0.067
Protein (g/1,000 kcal)	0.35	0.125
Fiber (g/1,000 kcal)	-0.09	0.690
Fiber – total intake (g)	-0.16	0.487
High fiber (%)		
**Physical activity**		
Sedentary time (min)	**0.54**	**0.012**
Light PA (min)	**-0.54**	**0.011**
MVPA (min)	-0.30	0.194
**Anthropometrics and body composition**		
Body weight (kg)	**0.68**	**0.001**
BMI z-scores	0.37	0.104
%BF (%)	0.41	0.065
FMI (kg/m^2)	**0.54**	**0.013**
FFMI (kg/m^2)	**0.64**	**0.002**
**Metabolic parameters**		
Glucose (mg/dL)	0.43	0.054
Insulin (pmol/L)	**0.98**	**>0.001**
CRP (mg/L)	0.21	0.367
IL-6 (pg/mL)	-0.21	0.381
TNF-α (pg/mL)	-0.18	0.464
LBP (ug/mL)	0.13	0.585
LPS (EU/mL)	0.10	0.673
**SCFAs***		
Acetic (mmol/kg)	-0.11	0.633
Propionic (mmol/kg)	0.12	0.598
Isobutyric (mmol/kg)	-0.14	0.548
Butyric (mmol/kg)	-0.21	0.352
Isovaleric (mmol/kg)	-0.10	0.678
Valeric (mmol/kg)	0.20	0.397
Total SCFAs (mmol/kg)	-0.08	0.741
**Ratios**		
F/B ratio	-0.19	0.409
P/B ratio	0.00	0.986

Abbreviations: %BF, percent body fat; CHO, carbohydrate; F/B ratio, Firmicutes to Bacteroidetes ratio; FFMI, fat-free mass index; FMI, fat mass index; HOMA-IR, homeostatic model assessment of insulin resistance; CRP, C-reactive protein; IL-6, interleukin-6; LBP, lipopolysaccharide binding protein; LPS, lipopolysaccharides; MVPA, moderate-to-vigorous physical activity; PA, physical activity; P/B, Prevotella to Bacteroides ratio SCFAs, short-chain fatty acids; TEI, total energy intake; TNF-α, tumor necrosis factor alpha. *Short-chain fatty acids (SCFAs) were normalized to the amount of input material (SCFA [in mmol] / fecal content [in kg]).

Supplementary Material

Table S3. Partial correlations adjusted for age between the homeostatic model assessment of insulin resistance (HOMA-IR) and α-diversity, species, and pathways (N=21).

**Partial correlation adjusted for Age**	**rho**	***P* value**
**Measures of α-diversity**		
Species richness	-0.283	0.227
Firmicutes richness	-0.121	0.61
Proteobacteria Shannon diversity	-0.589	**0.006**
Proteobacteria richness	-0.481	**0.032**
**Species**		
Oscillibacter sp CAG 241	-0.592	**0.006**
Haemophilus parainfluenzae	-0.445	**0.049**
Veillonella parvula	-0.37	0.108
Dialister invisus	-0.113	0.636
Agathobaculum butyriciproducens	-0.12	0.615
**Pathways**		
GLUTORN-PWY: L-ornithine biosynthesis	-0.594	**0.006**
PWY-3841: folate transformations II	-0.406	0.076
PWY4LZ-257: superpathway of fermentation (Chlamydomonas reinhardtii)	-0.435	0.055
PWY-7332: superpathway of UDP-N-acetylglucosamine-derived O-antigen building blocks biosynthesis	-0.486	**0.030**
P161-PWY: acetylene degradation	-0.417	0.067
COA-PWY-1: coenzyme A biosynthesis II (mammalian)	-0.374	0.104
PWY0-1298: superpathway of pyrimidine deoxyribonucleosides degradation	-0.465	**0.039**
OANTIGEN-PWY: O-antigen building blocks biosynthesis (E. coli)	-0.456	**0.043**
PWY-7400: L-arginine biosynthesis IV (archaebacteria)	-0.522	**0.018**
UDPNAGSYN-PWY: UDP-N-acetyl-D-glucosamine biosynthesis I	-0.458	**0.042**
P441-PWY: superpathway of N-acetylneuraminate degradation	-0.348	0.132
ARGSYN-PWY: L-arginine biosynthesis I (via L-ornithine)	-0.52	**0.019**
PWY-6549: L-glutamine biosynthesis III	-0.392	0.088
PWY0-1296: purine ribonucleosides degradation	-0.532	**0.016**
PWY-5384: sucrose degradation IV (sucrose phosphorylase)	-0.172	0.469
PWY-6471: peptidoglycan biosynthesis IV (Enterococcus faecium)	0.187	0.431
PWY-6470: peptidoglycan biosynthesis V (β-lactam resistance)	0.158	0.506
PWY-6628: superpathway of L-phenylalanine biosynthesis	-0.082	0.732

Table S4. Significant contributors of bacterial species β-diversity detected by canonical correspondence analysis (CCA) (N = 21).

Species	Mean abundance (%)	CCA1	CCA2	rho	*P* value
Prevotella copri	28.97	1.00	-0.09	0.99	0.001
Paraprevotella xylaniphila	0.03	0.70	0.72	0.50	0.002
Lachnospira pectinoschiza	1.18	-0.91	-0.41	0.41	0.007
Paraprevotella clara	0.00	0.09	1.00	0.91	0.011
Faecalibacterium prausnitzii	8.64	-0.99	0.12	0.42	0.012
Ruminococcus bicirculans	1.54	-0.96	-0.26	0.37	0.013
Roseburia sp CAG 471	0.01	0.17	0.99	0.79	0.015
Firmicutes bacterium CAG 95	0.18	0.07	1.00	0.91	0.021
Roseburia sp CAG 309	0.01	0.00	1.00	0.86	0.021
Fusicatenibacter saccharivorans	0.58	-1.00	0.00	0.42	0.021
Alistipes indistinctus	0.07	0.10	1.00	0.90	0.022
Butyricimonas virosa	0.12	-0.11	0.99	0.48	0.022
Bacteroides ovatus	1.54	-1.00	-0.05	0.43	0.025
Agathobaculum butyriciproducens	0.09	-0.30	0.95	0.53	0.026
Eubacterium sp CAG 180	1.77	-0.01	1.00	0.55	0.027
Bacteroides vulgatus	10.37	-0.85	-0.53	0.35	0.027
Bacteroides xylanisolvens	0.49	-0.28	0.96	0.40	0.029
Bacteroides uniformis	7.42	-1.00	-0.02	0.48	0.039
Odoribacter splanchnicus	0.53	-0.32	0.95	0.37	0.039
Bacteroides coprocola	0.05	0.07	1.00	0.91	0.046
Prevotella sp CAG 279	0.53	0.07	1.00	0.91	0.046
Prevotella sp CAG 873	0.16	0.07	1.00	0.91	0.046
Ruminococcus champanellensis	0.00	0.07	1.00	0.91	0.046
Ruminococcus sp CAG 488	0.10	0.07	1.00	0.91	0.046
Firmicutes bacterium CAG 238	0.06	0.07	1.00	0.91	0.046
Firmicutes bacterium CAG 534	0.02	0.07	1.00	0.91	0.046
Klebsiella michiganensis	0.00	0.07	1.00	0.91	0.046
Klebsiella pneumoniae	0.01	0.07	1.00	0.91	0.046
Klebsiella variicola	0.00	0.07	1.00	0.91	0.046
Blastocystis sp subtype 1	0.00	0.07	1.00	0.91	0.046

Abbreviations: CCA1, canonical correspondence analysis axis 1; CCA2, canonical correspondence analysis axis 2.

Supplementary Material

2 Supplementary Figures

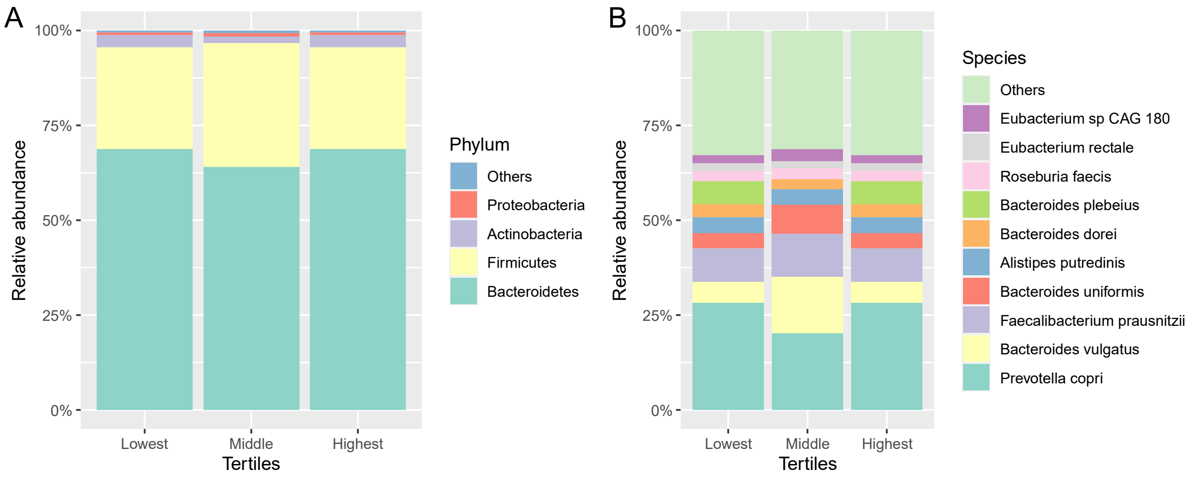

Figure S1. Relative abundance of A) phyla and B) species across homeostatic model assessment of insulin resistance (HOMA-IR) tertiles. Note that data are graphically represented using mean abundance values; however, there were no significant differences in the median abundance of phyla and species between tertiles in non-parametric analyses (i.e., Dunn's test with Benjamin-Hochberg correction).

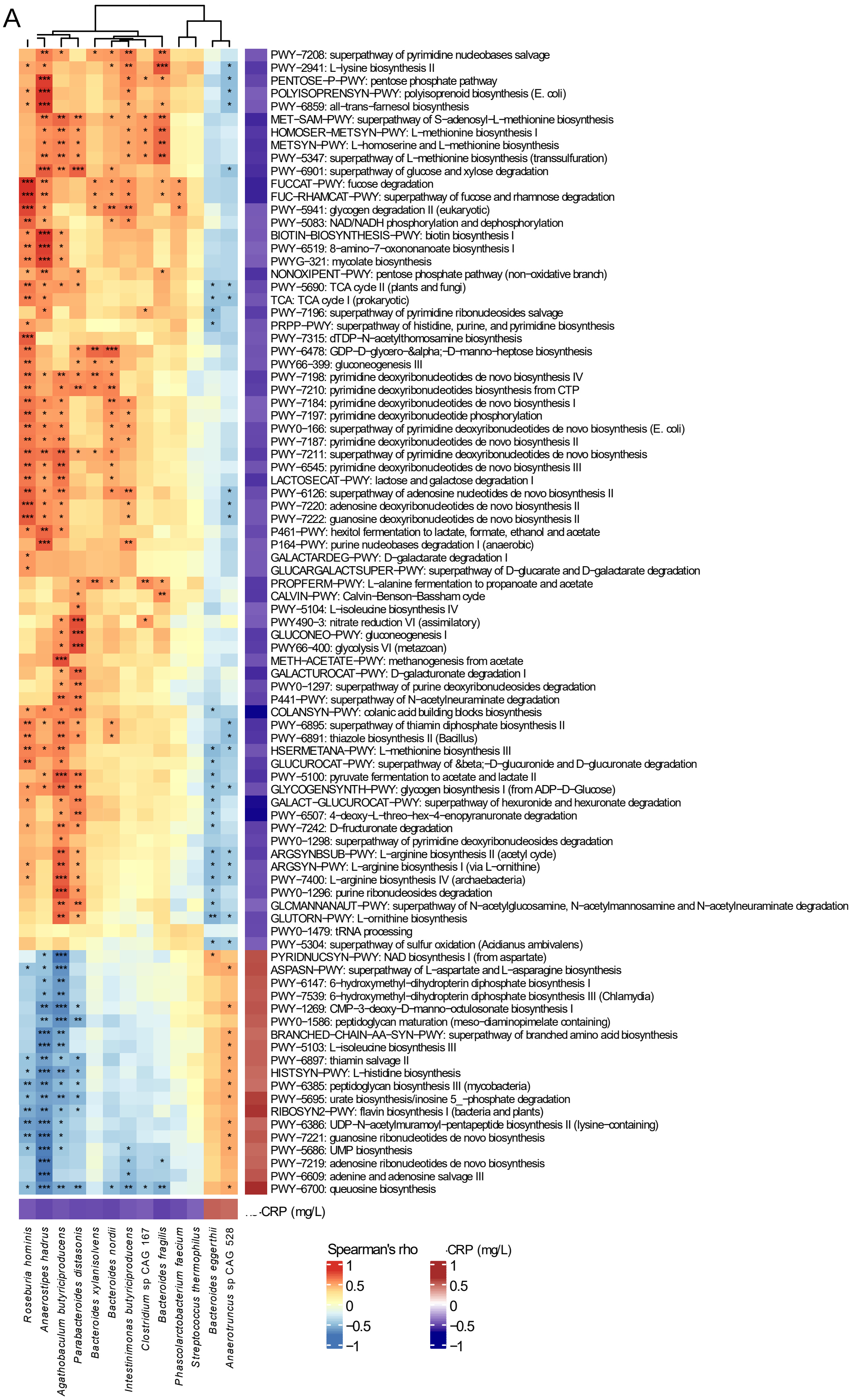
A
PWY−7208: superpathway of pyrimidine nucleobases salvage
PWY−2941: L−lysine biosynthesis II
PENTOSE−P−PWY: pentose phosphate pathway
POLYISOPRENSYN−PWY: polyisoprenoid biosynthesis (E. coli)
PWY−6859: all−trans−farnesol biosynthesis
MET−SAM−PWY: superpathway of S−adenosyl−L−methionine biosynthesis
HOMOSER−METSYN−PWY: L−methionine biosynthesis I
METSYN−PWY: L−homoserine and L−methionine biosynthesis
PWY−5347: superpathway of L−methionine biosynthesis (transsulfuration)
PWY−6901: superpathway of glucose and xylose degradation
FUCCAT−PWY: fucose degradation
FUC−RHAMCAT−PWY: superpathway of fucose and rhamnose degradation
PWY−5941: glycogen degradation II (eukaryotic)
PWY−5083: NAD/NADH phosphorylation and dephosphorylation
BIOTIN−BIOSYNTHESIS−PWY: biotin biosynthesis I
PWY−6519: 8−amino−7−oxononanoate biosynthesis I
PWYG−321: mycolate biosynthesis
NONOXIPENT−PWY: pentose phosphate pathway (non−oxidative branch)
PWY−5690: TCA cycle II (plants and fungi)
TCA: TCA cycle I (prokaryotic)
PWY−7196: superpathway of pyrimidine ribonucleosides salvage
PRPP−PWY: superpathway of histidine, purine, and pyrimidine biosynthesis
PWY−7315: dTDP−N−acetylthomosamine biosynthesis
PWY−6478: GDP−D−glycero−α−D−manno−heptose biosynthesis
PWY66−399: gluconeogenesis III
PWY−7198: pyrimidine deoxyribonucleotides de novo biosynthesis IV
PWY−7210: pyrimidine deoxyribonucleotides biosynthesis from CTP
PWY−7184: pyrimidine deoxyribonucleotides de novo biosynthesis I
PWY−7197: pyrimidine deoxyribonucleotide phosphorylation
PWY0−166: superpathway of pyrimidine deoxyribonucleotides de novo biosynthesis (E. coli)
PWY−7187: pyrimidine deoxyribonucleotides de novo biosynthesis II
PWY−7211: superpathway of pyrimidine deoxyribonucleotides de novo biosynthesis
PWY−6545: pyrimidine deoxyribonucleotides de novo biosynthesis III
LACTOSECAT−PWY: lactose and galactose degradation I
PWY−6126: superpathway of adenosine nucleotides de novo biosynthesis II
PWY−7220: adenosine deoxyribonucleotides de novo biosynthesis II
PWY−7222: guanosine deoxyribonucleotides de novo biosynthesis II
P461−PWY: hexitol fermentation to lactate, formate, ethanol and acetate
P164−PWY: purine nucleobases degradation I (anaerobic)
GALACTARDEG−PWY: D−galactarate degradation I
GLUCARGALACTSUPER−PWY: superpathway of D−glucarate and D−galactarate degradation
PROPFERM−PWY: L−alanine fermentation to propanoate and acetate
CALVIN−PWY: Calvin−Benson−Bassham cycle
PWY−5104: L−isoleucine biosynthesis IV
PWY490−3: nitrate reduction VI (assimilatory)
GLUCONEO−PWY: gluconeogenesis I
PWY66−400: glycolysis VI (metazoan)
METH−ACETATE−PWY: methanogenesis from acetate
GALACTUROCAT−PWY: D−galacturonate degradation I
PWY0−1297: superpathway of purine deoxyribonucleosides degradation
P441−PWY: superpathway of N−acetylneuraminate degradation
COLANSYN−PWY: colanic acid building blocks biosynthesis
PWY−6895: superpathway of thiamin diphosphate biosynthesis II
PWY−6891: thiazole biosynthesis II (Bacillus)
HSERMETANA−PWY: L−methionine biosynthesis III
GLUCUROCAT−PWY: superpathway of β−D−glucuronide and D−glucuronate degradation
PWY−5100: pyruvate fermentation to acetate and lactate II
GLYCOGENSYNTH−PWY: glycogen biosynthesis I (from ADP−D−Glucose)
GALACT−GLUCUROCAT−PWY: superpathway of hexuronide and hexuronate degradation
PWY−6507: 4−deoxy−L−threo−hex−4−enopyranuronate degradation
PWY−7242: D−fructuronate degradation
PWY0−1298: superpathway of pyrimidine deoxyribonucleosides degradation
ARGSYNBSUB−PWY: L−arginine biosynthesis II (acetyl cycle)
ARGSYN−PWY: L−arginine biosynthesis I (via L−ornithine)
PWY−7400: L−arginine biosynthesis IV (archaebacteria)
PWY0−1296: purine ribonucleosides degradation
GLCMANNANAUT−PWY: superpathway of N−acetylglucosamine, N−acetylmannosamine and N−acetylneuraminate degradation
GLUTORN−PWY: L−ornithine biosynthesis
PWY0−1479: tRNA processing
PWY−5304: superpathway of sulfur oxidation (Acidianus ambivalens)
PYRIDNUCSYN−PWY: NAD biosynthesis I (from aspartate)
ASPASN−PWY: superpathway of L−aspartate and L−asparagine biosynthesis
PWY−6147: 6−hydroxymethyl−dihydropterin diphosphate biosynthesis I
PWY−7539: 6−hydroxymethyl−dihydropterin diphosphate biosynthesis III (Chlamydia)
PWY−1269: CMP−3−deoxy−D−manno−octulosonate biosynthesis I
PWY0−1586: peptidoglycan maturation (meso−diaminopimelate containing)
BRANCHED−CHAIN−AA−SYN−PWY: superpathway of branched amino acid biosynthesis
PWY−5103: L−isoleucine biosynthesis III
PWY−6897: thiamin salvage II
HISTSYN−PWY: L−histidine biosynthesis
PWY−6385: peptidoglycan biosynthesis III (mycobacteria)
PWY−5695: urate biosynthesis/inosine 5_−phosphate degradation
RIBOSYN2−PWY: flavin biosynthesis I (bacteria and plants)
PWY−6386: UDP−N−acetylmuramoyl−pentapeptide biosynthesis II (lysine−containing)
PWY−7221: guanosine ribonucleotides de novo biosynthesis
PWY−5686: UMP biosynthesis
PWY−7219: adenosine ribonucleotides de novo biosynthesis
PWY−6609: adenine and adenosine salvage III
PWY−6700: queuosine biosynthesis
CRP (mg/L)
Roseburia hominis
Anaerostipes hadrus
Agathobaculum butyriciproducens
Parabacteroides distasonis
Bacteroides xylanisolvens
Bacteroides nordii
Intestinimonas butyriciproducens
Clostridium sp CAG 167
Bacteroides fragilis
Phascolarctobacterium faecium
Streptococcus thermophilus
Bacteroides eggerthii
Anaerotruncus sp CAG 528
Spearman's rho
1
0.5
0
−0.5
−1
CRP (mg/L)
1
0.5
0
−0.5
−1

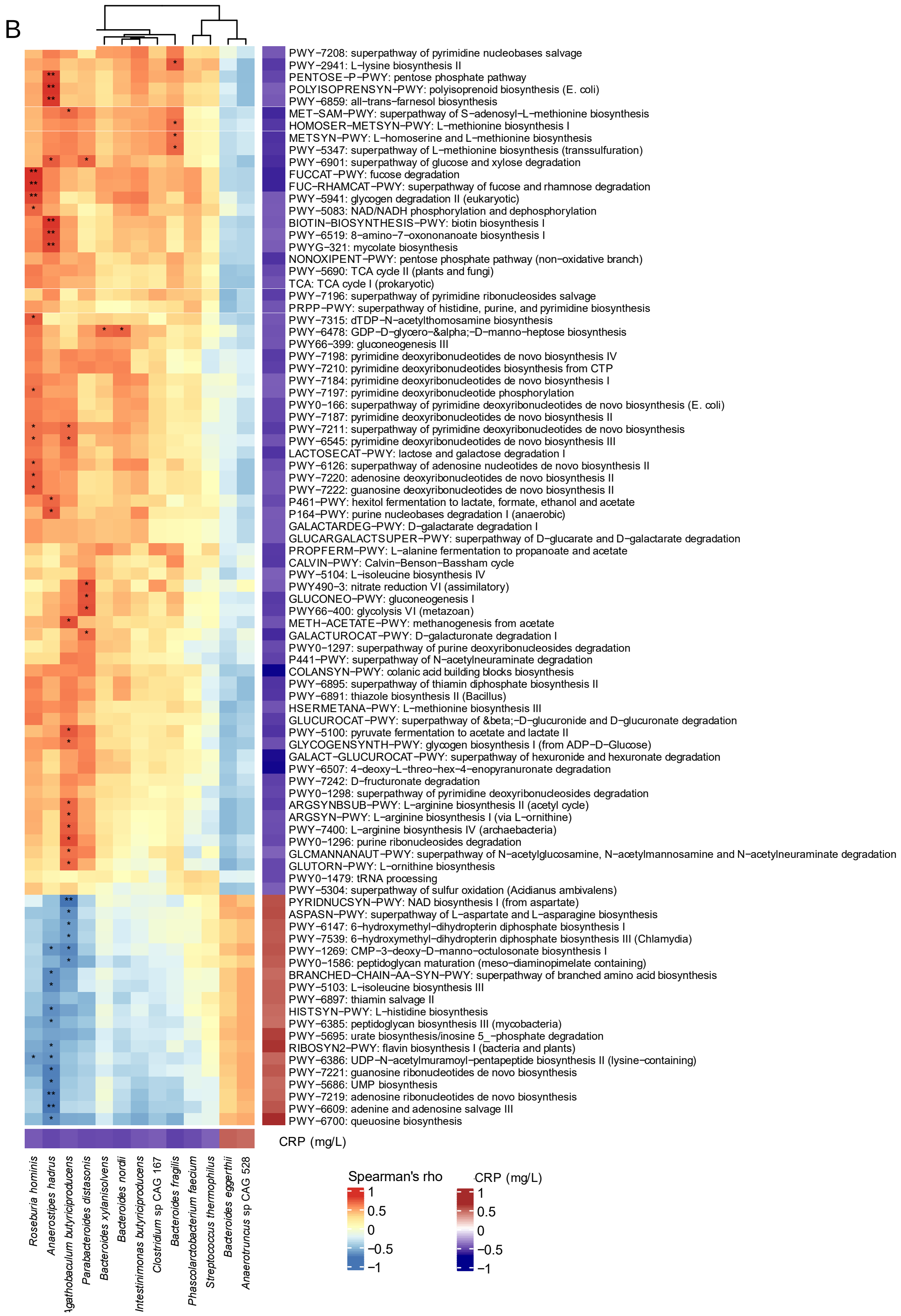

Figure S2. Heat maps showing correlations of gut microbiota composition and function with C-reactive protein (CRP) in 21 children with obesity. A) uncorrected correlations and B) corrected correlations using the Benjamin-Hochberg method. * $p < 0.05$; ** $p < 0.01$; *** $p < 0.001$.

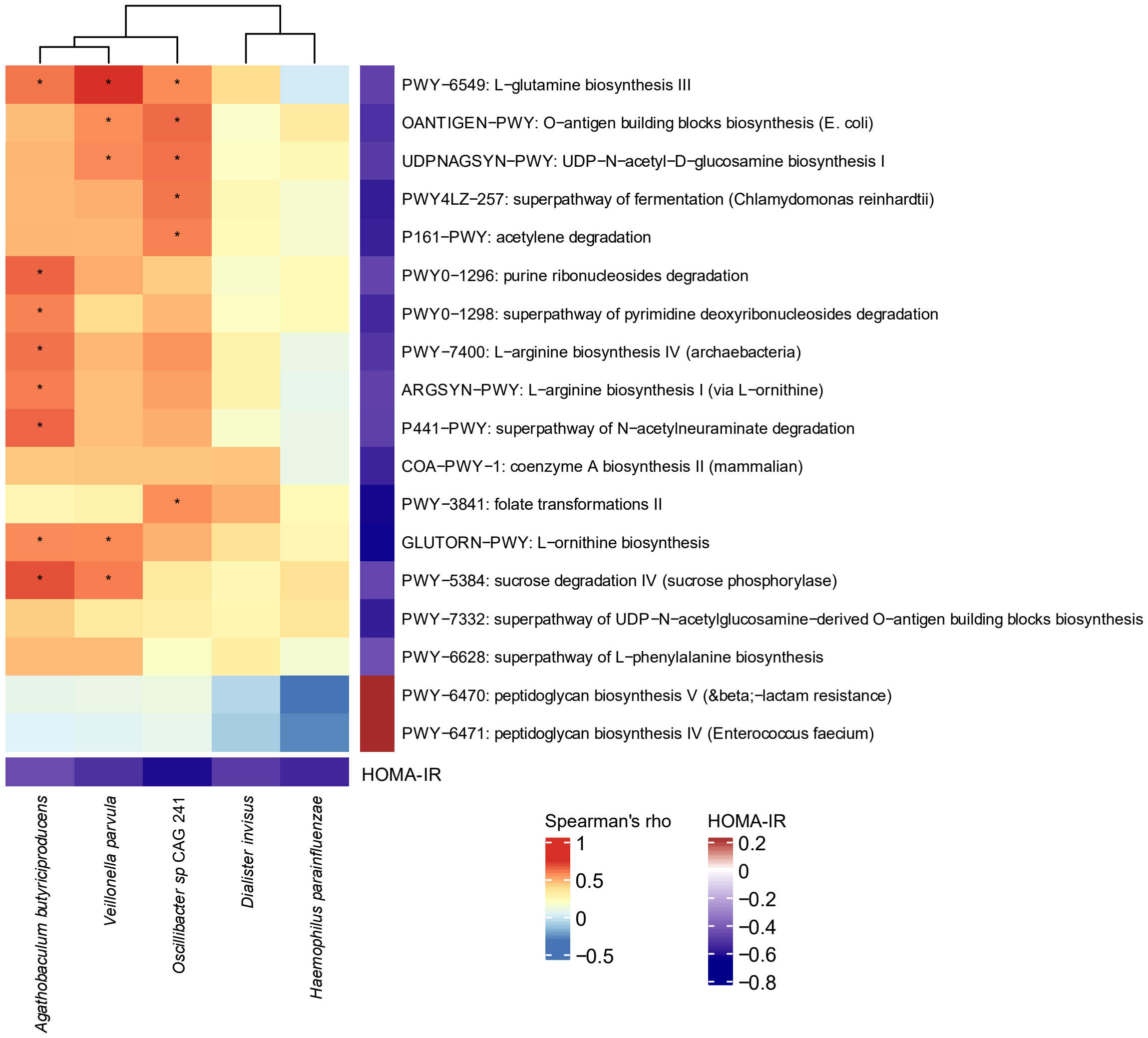

Figure S3. Heat map showing correlations of gut microbiota composition and function with the homeostatic model assessment for insulin resistance (HOMA-IR) in 21 children with obesity (p values corrected using the Benjamin-Hochberg method). * $p < 0.05$; ** $p < 0.01$; *** $p < 0.001$.

Supplementary Material

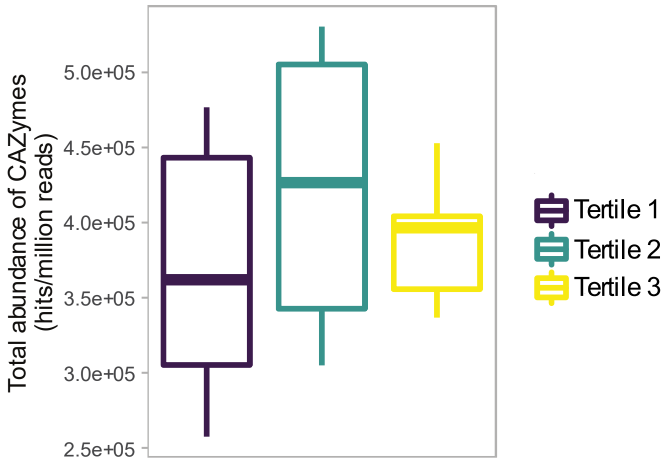

Figure S4. Abundance of Carbohydrate-Active Enzyme (CAZyme) in children with obesity stratified by data-driven HOMA-IR tertiles. Comparisons between HOMA-IR tertiles were performed using the Dunn's test with Benjamin-Hochberg correction.
